# The combined impact of social networks and connectedness on anxiety, stress, and depression during COVID-19 quarantine: a retrospective observational study

**DOI:** 10.3389/fpubh.2023.1298693

**Published:** 2023-12-19

**Authors:** Huiting Luo, Dan Luo, Qiao Tang, Zhiang Niu, Jiajun Xu, Jing Li

**Affiliations:** Mental Health Center, West China Hospital, Sichuan University, Chengdu, China

**Keywords:** depression, anxiety, social isolation, moderated mediation, COVID-19, social connectedness

## Abstract

**Introduction:**

The COVID-19 pandemic and associated quarantine measures have precipitated a surge in mental health disorders, particularly depression and anxiety. Government policies and restrictions on physical activity have contributed to this phenomenon, as well as diminished subjective social connectedness and exacerbated objective social isolation. As two dimensions of social isolation, it is worth noting that subjectively perceived social connectedness serves as a protective factor for mental health, whereas the decline in the size of objectively evaluated social networks poses a significant risk. However, research investigating the combined influence of these two dimensions remains limited.

**Methods:**

This study used an online survey to collect data to investigate the effects of objective social connectedness and objective social networks on anxiety, stress, and depression during COVID-19 quarantine. A total of 485 participants were analyzed using statistical methods, including paired *t*-test, Pearson correlation analysis, linear regression, cluster analysis, ANOVA, and moderated mediated.

**Results:**

The study found that anxiety and depression scores increased during the quarantine, with age, education, and social connectedness scores associated with the increase. Pre-quarantine anxiety and depression levels were strongly correlated with mental health status during quarantine. Cluster analysis, respectively, revealed three clusters for those without increasing anxiety and depression scores. The study also found that objective social network influences the impact of subjective social connectedness on pre-quarantine mental health, which in turn affects anxiety and depression levels during quarantine.

**Conclusion:**

The study identified that quarantine increased anxiety and depression, with age being protective, and education and subjective social connectedness as risk factors. The study also emphasizes the comprehensive impact of objective and subjective social isolation. Although individuals perceive the same degree of social connectedness, those with smaller social networks are more prone to developing symptoms of anxiety and depression, which are also more likely to worsen during quarantine.

## 1 Introduction

The emergence of the COVID-19 pandemic in December 2019, which caused severe health implications and fatalities among those infected (1), has also had a profound impact on global mental health (2). Before the pandemic, the prevalence of major depressive disorder and anxiety disorders was 2.2% was 3.3% in China (3). People perceived stress in their daily lives, primarily including work pressures, school pressures, and family responsibilities. However, a staggering 27.6% increase in depression disorders and a 25.6% increase in anxiety disorders have been observed worldwide post the 2020 epidemic (4). Additionally, the COVID-19 pandemic created an environment that encompasses social restrictions, quarantine measures, closures of schools and businesses, loss of livelihoods, and reduced economic activity, which may severely impact the population’s mental well-being (5). Economic uncertainties and disruptions became the new norm, intensifying anxiety. The inability to engage in usual social activities, coupled with fears of infection and unemployment, exacerbated stress levels. Quarantine and maintained social and physical distance can limit social interaction, triggering a host of negative consequences such as boredom, depression, feeling burdened, loneliness, and fear. These, in turn, pose risk factors for mental health problems, including anxiety, depression, suicide, and self-harm (6). Moreover, isolation measures coupled with a lack of interpersonal communication may exacerbate depression (7). Studies have suggested a major infectious disease can have many psychological effects on the public, which can be expressed as anxiety, fear, and worry (8). A cross-sectional study in China during the pandemic found prevalence rates of depression and anxiety of 20.1 and 18.2% (9). In these cases, it is necessary to safeguard the mental health of isolated populations and to identify and implement strategies and actions to promote mental health (10).

Social isolation consists of two separate structures, subjective social isolation, and objective social isolation (11, 12). Social connectedness, as subjective social isolation, is an important protective factor for psychosocial adjustment. It refers to an individual’s subjective perception of maintaining close interpersonal relationships with society, including relationships with family, friends, community, school, and neighborhood (13). When individuals experience high levels of social connectedness, they tend to encounter a decrease in anxiety and depression, an increase in self-esteem, and an improvement in general population health (14). Conversely, individuals who lack social connectedness experience a pervasive sense of isolation and loneliness, which can be detrimental to their mental health (15). Reduced activity space and economic instability due to quarantine can increase loneliness (16). Research has found that social connectedness is associated with decreased anxiety, loneliness, suicidal ideation or attempts, and depression (17). An increase in social connectedness during the ongoing COVID-19 pandemic also has been shown to ameliorate mental health indicators (18).

Objective social isolation, measured by assessing the size of one’s social network, is characterized by the lack or limitation of close personal contact with friends, family, and community ties (19, 20). The COVID-19 pandemic caused the death of family and friends, increased unemployment, and quarantine leading to a decrease in the activities of daily living, and these changes brought a reduction in the size of social networks (21). It has emerged as a growing public health problem with serious implications for both physical and mental health. Indeed, objective social isolation may be considered an indication of compromised health and an unfavorable prognosis (22). The literature highlights that objective social isolation stands as a significant risk factor for diminished well-being, cognitive degeneration, and mortality within the general populace (23, 24). Social network is also associated with poor physical health and mental illnesses such as depressive symptoms (25, 26).

Objective social isolation limits opportunities for social interaction and may lead to feelings of loneliness, low self-esteem, and depression. Studies have found that higher loneliness scores are associated with lower levels of social connectedness (27). However, individuals experiencing smaller social networks may not necessarily feel subjective social isolation (28). Compared to males, some females may still perceive lower social connectedness despite having larger social network sizes (29). Older adults are particularly at risk for objective social isolation and subjective loneliness because of retirement, deaths of family members and friends, and declining quality of life (30). Based on our research, there have been limited previous studies that integrated both subjective and objective dimensions of social isolation to explore the impact on mental health. Therefore, the study was designed to explore changes in anxiety, stress, and depression as a result of experiencing quarantine, and simultaneously explore the combined impact of subjective social connectedness and objective social networks on these changes. In addition, we hypothesized that objective social networks would moderate the effect of subjective social connectedness on mental health status during the quarantine.

## 2 Methods

### 2.1 Participants

This study was completed from September 2022 to October 2022 in Chengdu, Sichuan Province, China, where all participants were undergoing a city-wide quarantine during the COVID-19 pandemic. All procedures were conducted in accordance with the ethical standards of the 1964 Declaration of Helsinki and its later amendments. The study was approved by the West China Hospital of Sichuan University Biomedical Research Ethics Committee on February 24, 2020 [Ethics Number: K2020006]. Retrospective data collection was based on an online web-based survey that collected demographic data, social networks, changes in anxiety, stress, and depression levels, and social connectedness of participants before and during the city-wide quarantine. All participants read informed consent before completing the questionnaire and were informed of the purpose of the study, the methods, and the risks and benefits they might derive from the study. Inclusion criteria: consent to participate in this questionnaire; quarantine status when completing the questionnaire. Exclusion criteria: previous diagnosis of novel coronavirus pneumonia; previous diagnosis of mental disorder. Because their mental health may be particularly vulnerable to the pandemic (31). Studies from different countries have found that individuals reporting COVID-19 symptoms, as well as those convalescing from acute COVID-19 illness exhibit higher levels of anxiety, depression, suicidal ideation, loneliness, and quality of life compared to healthy individuals (32–40). A total of 519 participants completed the survey. Finally, 485 participants were retained.

### 2.2 Materials

#### 2.2.1 Depression, anxiety and stress scale—Short form (DASS-21)

The DASS-21, a reliable and validated instrument, finds application within both clinical and non-clinical adult populations. Its purpose is to assess the perceived intensity of symptoms associated with depression, anxiety, and stress (41–43). It contains 21 items, with 7 items per subscale, answered on a four-point scale ranging from 0 (does not apply to me) to 3 (applies to me or most of the time), considering the extent to which each item has been applied to them in the past week. Standardized scores were the raw score multiplied by 2. Depressive symptoms were classified according to the following ranges: mild (10–15), moderate (14–20), severe (21–27), and extremely severe (≥28). Analogously, anxiety symptomatology fell into categories of mild (8, 9), moderate (10–14), severe (15–19), and extremely severe (≥20). Stress levels were assessed across the ranges of mild (15–18), moderate (19–25), severe (26–33), and extremely severe (≥34). The DASS-21 scale has good reliability and validity in mainland China and can be used as a valid tool for the evaluation of depression-anxiety-stress in adult residents (44). In our sample, before and after quarantine, the Cronbach’s alpha coefficients for the total DASS-21 scale were 0.951/0.968, and for the three subscales of stress, anxiety, and depression, they were 0.887/0.926, 0.851/0.905, and 0.885/0.922, respectively.

#### 2.2.2 Social connectedness scale-revised (SCS-R)

The SCS-R is a self-report scale consisting of 20 items that assess the degree to which individuals experience both intimacy and separation in interpersonal relationships (45, 46). Participants rated items using a six-point Likert scale ranging from 1 (strongly disagree) to 6 (strongly agree). The higher the score, the higher the degree of social connectedness, with an internal consistency coefficient of 0.916 and retest reliability of 0.845 for the Chinese version (47). Cronbach alpha coefficients for the SCS-R were 0.792/0.809 in our study.

#### 2.2.3 Berkman-Syme social network index (SNI)

The SNI can be used to measure the objective social isolation in China (48, 49). The scale assesses social networks and has four self-reported subcomponents: married (no = 0; yes = 1); close friends and relatives (0–2 friends and 0–2 relatives = 0, all other scores = 1); group involvement (no = 0; yes = 1); and participation in religious gatherings or community service (≤every few months = 0; >once or twice a month = 1). Scores range from 0 to 4, with lower scores representing smaller social networks. Based on previous scoring conventions, scores of 0 and 1 were categorized as the most socially isolated (22).

### 2.3 Statistical analysis

We used absolute skewness values greater than 2 or absolute kurtosis (appropriate value) greater than 7 as reference values for determining severe non-normality (50). Continuous variables with normal distributions were presented using mean ± standard deviation and non-normal distributions were presented using median (interquartile range). N (%) was used for categorical variables. Correlations of normally distributed continuous variables were analyzed using Pearson correlation analysis. Univariate linear regression was used to identify factors potentially associated with a change in anxiety scores (anxiety.d.) and change in depression scores (depression.d.) during the quarantine. These factors with *p* < 0.1 were entered into a multiple linear regression to examine the effects of pre-quarantine levels of social connectedness (SCS_R.B.) and social networks (SNI) on anxiety and depression score changes (51).

After centering all continuous variables, the moderated mediating effects were assessed using Model 7 (the model assumes that the first half of the mediating path is moderated by the moderating variable) proposed by Hayes (52). Statistical analyses were performed using SPSS Statistics for Mac 26.0 and the “PROCESS” macro in SPSS (version 4.2).

Cluster analyses were performed using R (v4.1.2) to subdivide those whose anxiety and depressive symptoms do not worsen during quarantine. Pre-cluster evaluation and calculation of silhouette width were performed using the R package Factoextra (v1.0.7), using the Hopkins statistics to check whether the data has clusterable features and using the average silhouette width to evaluate the clustering effect (53). The final value of this indicator is between −1 and 1. The closer to 1, the more accurate the sample classification, and the less than 0, the sample may be misclassified. K-means cluster analyses were performed using the R package Cluster (v2.1.4) and ggplot2 (v3.4.1). Comparison of means between the three clusters after cluster analysis was performed by ANOVA and multiple comparison analysis using Scheffe’s test due to the unequal sample sizes of the clusters.

## 3 Results

### 3.1 Demographic characteristics

In this study, 485 participants were eventually included in the final data analysis (Table 1). The majority of participants were female (69.28%), married status (56.49%), had a full-time job (67.62%), and had a monthly income >5,000 (64.33%). The primary quarantine mode in this quarantine was quarantine at home, and the median number of days of quarantine and the number of previous quarantines were 11 days and 2 times, respectively. The mean of the SNI was 1.67, which did not belong to the most socially isolated (SNI = 0/1).

**Table 1:** Characteristics of study participants (*N* = 485).

Characteristic	Value
Age	36.97 ± 13.35
Gender	
Female	336 (69.28%)
Education	15.89 ± 3.99
Marital status	
Married	274 (56.49%)
Employment state	
Full-time	328 (67.62%)
Monthly income (RMB)*	
> 5,000	312 (64.33%)
Smoking	
Yes	63 (12.99%)
Drinking	
Yes	45 (9.28%)
Physical Disorders	
Yes	35 (7.22%)
Quarantine alone	
No	389 (80.21%)
Quarantine mode	
Quarantine at home	428 (88.25%)
The number of quarantine experiences	2 (1–2)
Current number of days in quarantine	11 (10–12)
SNI	1.67 ± 1.01

Values are *n* (%), mean ± standard deviation or median (interquartile range). *1 RMB was equal to approximately 0.14 USD at the time of the study. SNI=Berkman-Syme social network index.

### 3.2 The correlation analysis between mental state and the level of social connectedness and social isolation before and during the quarantine

**Figure 1:**
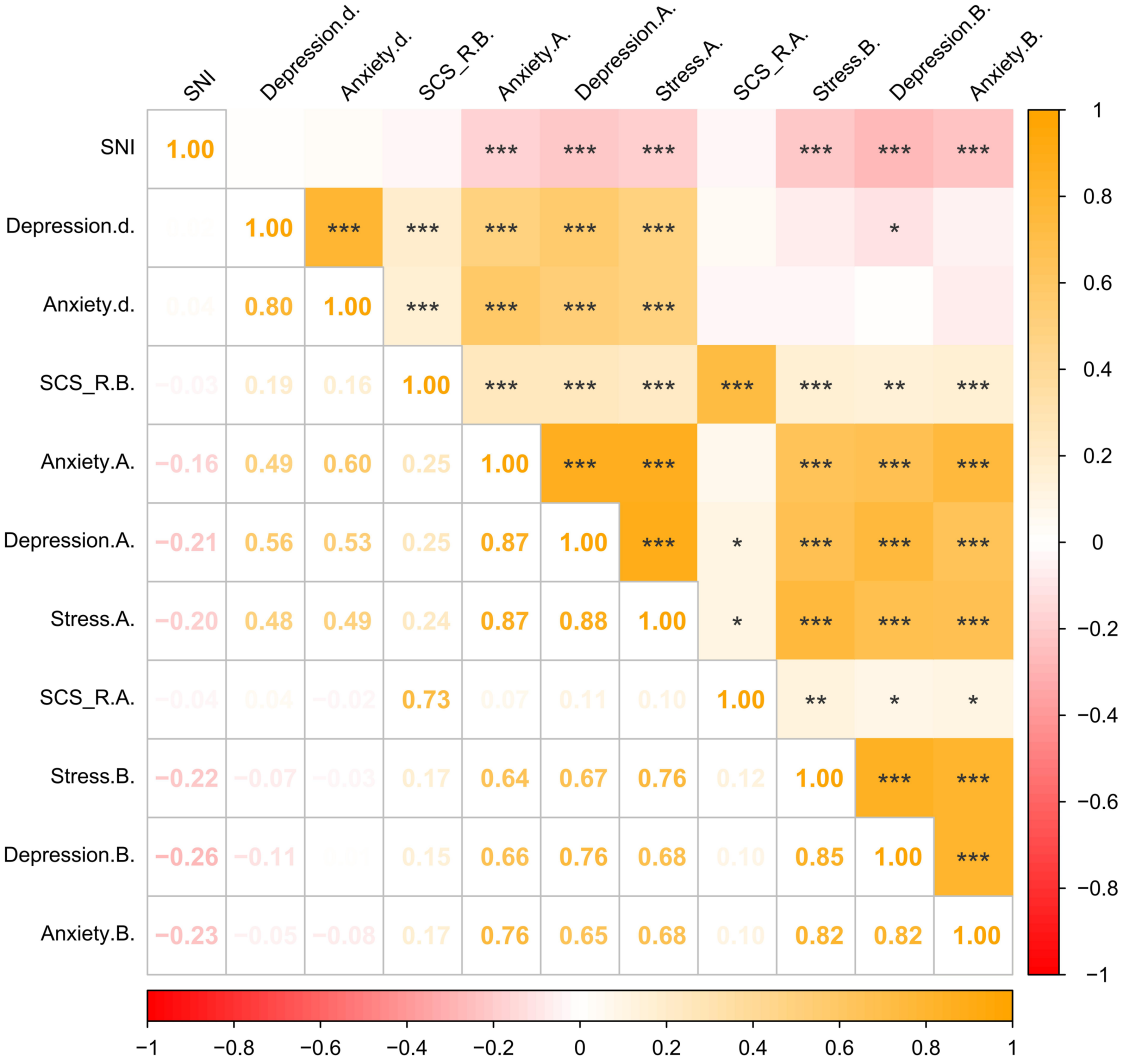
Correlation matrix of the main variables (*N* = 485). *p < 0.05, **p < 0.01, ***p < 0.001. SNI = Berkman-Syme social network index; Depression.d. = Change in Depression Score During the Quarantine minus Pre-quarantine Depression Score; Anxiety. d. = Change in Anxiety Score During the Quarantine minus Pre-quarantine Anxiety Score; SCS_R.B. = Pre-quarantine Levels of Social Connectedness Scale-Revised; Anxiety. A. = Anxiety Score During the Quarantine; Depression. A. = Depression Score During the Quarantine; Stress. A. = Stress Score During the Quarantine; SCS_R.A. = Levels of Social Connectedness Scale-Revised During the Quarantine; Anxiety. B.= Pre-quarantine Anxiety Score; Depression.B. = Pre-quarantine Depression Score; Stress. B. = Pre-quarantine Stress Score.

After paired (*t*-tests, we found that anxiety (*t* = 2.605, *p* = 0.009) and depression (*t* = 2.302, *p =* 0.022) scores increased during the quarantine period compared to the pre-quarantine period, while stress scores and social connectedness scores before and during the quarantine did not show significant differences (Supplementary Table S1). Subsequently, performing correlation analysis revealed that the pre-quarantine levels of anxiety and depression were strongly correlated with the levels of anxiety and depression during the quarantine period. In addition, pre-quarantine anxiety and depression scores were positively correlated with pre-quarantine social connectedness scores (*r =* 0.17, *p* < 0.001; *r =* 0.15, *p* < 0.01) and negatively correlated with SNI scores (*r =* −0.23, *p* < 0.001; *r =* −0.26, *p* < 0.001). Notably, the difference in anxiety and depression scores exhibited a positive correlation with the pre-quarantine social connectedness scores (*r =* 0.16, *p* < 0.001; *r =* 0.19, *p* < 0.001), implying that higher pre-quarantine social connectedness scores were associated with greater increases in anxiety and depression scores during quarantine (Figure 1).

### 3.3 Factors associated with changes in anxiety and depression scores before and during the quarantine

We found that age, Anxiety.B. significantly and negatively predicted the change in anxiety scores before and during the quarantine, and SCS_R.B. significantly and positively predicted the change in anxiety scores. Age, Depression.B. significantly and negatively predicted the change in depression scores before and during the quarantine, and SCS_R.B. and education significantly and positively predicted the change in depression scores (Table 2). We conducted a multiple covariance test and found that for both models for the change in anxiety scores and depression scores, all of the VIF values in the models were less than 5. This implies that neither model had a covariance problem. Additionally, the Durbin-Watson (D-W) values were 2.088 and 2.051, suggesting that the models were not autocorrelated. Consequently, there was no correlation detected among the sample data, indicating that the models were robust.

**Table 2:** Factors associated with changes in anxiety and depression scores before and during the quarantine.

Variables	Anxiety.d.				Depression.d.			
	Univariate		Multivariate		Univariate		Multivariate	
	*B* (95% CI)	*P*	*B* (95% CI)	*P*	*B* (95% CI)	*P*	*B* (95% CI)	*P*
Age	−0.035 (−0.072, 0.002)	0.061	−0.045* (−0.082, −0.008)	0.017*	−0.046* (−0.088, −0.003)	0.035*	−0.051 (−0.093, −0.008)	0.020*
Education	0.096 (−0.028, 0.219)	0.131			0.196** (0.055, 0.337)	0.007**	0.162 (0.021, 0.302)	0.024*
Gender	0.487 (−0.582, 1.557)	0.372			−0.222 (−1.448, 1.004)	0.723		
Marital status	−0.131 (−1.127, 0.865)	0.796			−0.855 (−1.994, 0.284)	0.142		
Employment state	0.544 (−0.632, 1.719)	0.365			0.406 (−1.009, 1.821)	0.574		
Monthly income (RMB)	0.049 (−0.982, 1.080)	0.926			−0.388 (−1.568, 0.793)	0.520		
Smoking	0.159 (−1.310, 1.627)	0.832			0.186 (−1.497, 1.868)	0.829		
Drinking	−0.625 (−2.326, 1.076)	0.472			−0.389 (−2.339, 1.561)	0.696		
Physical disorders	−0.645 (−2.552, 1.262)	0.508			−0.531 (−2.717, 1.655)	0.634		
Quarantine mode	−0.370 (−1.903, 1.163)	0.636			−0.600 (−2.355, 1.156)	0.504		
Quarantine alone	0.949 (−0.288, 2.185)	0.133			0.393 (−1.026, 1.813)	0.588		
The number of quarantine experiences	0.152 (−0.298, 0.603)	0.507			0.246 (−0.270, 0.762)	0.351		
Current number of days in quarantine	−0.030 (−0.158, 0.098)	0.643			−0.015 (−0.161, 0.132)	0.844		
SNI	0.202 (−0.286, 0.689)	0.418			0.112 (−0.447, 0.671)	0.695		
SCS_R.B.	0.078** (0.036,0.120)	<0.001**	0.088 (0.046,0.130)	<0.001**	0.103** (0.056, 0.151)	<0.001**	0.120 (0.072, 0.167)	<0.001**
Stress.B.	−0.019 (−0.075, 0.037)	0.511			−0.048 (−0.113, 0.016)	0.139		
Anxiety.B.	−0.063 (−0.135, 0.010)	0.090	−0.517 (−0.951, −0.084)	0.020*	−0.043 (−0.126, 0.040)	0.312		
Depression.B.	0.004 (−0.057, 0.065)	0.904			−0.087* (−0.156, −0.017)	0.015*	−0.120 (−0.190, −0.051)	0.001**

*p < 0.05 **p < 0.01. SNI=Berkman-Syme social network index; Anxiety.B. = pre-quarantine anxiety score; Depression.B. = pre-quarantine depression score; SCS_R.B. = pre-quarantine levels of social connectedness scale-revised; Boot95%CI = the lower and upper limits of the 95% interval for bootstrap sampling. Depression.d.=Change in Depression Score During the Quarantine minus Pre-quarantine Depression Score; Anxiety.d.=Change in Anxiety Score During the Quarantine minus Pre-quarantine Anxiety Score.

### 3.4 Cluster analysis of people whose anxiety and depression did not deteriorate during the quarantine

**Figure 2:**
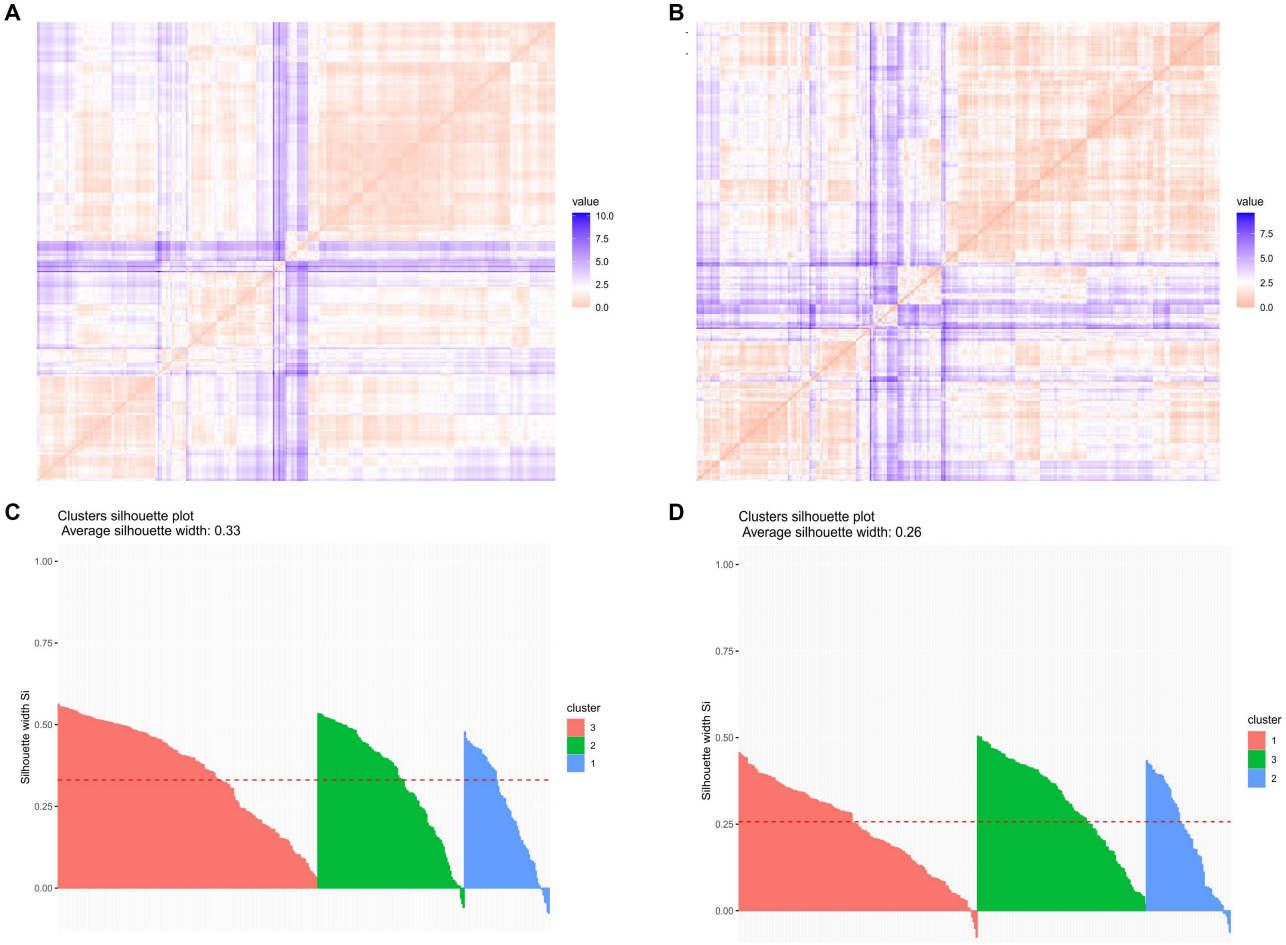
Analysis of clustering trends and clustering effects. (A) Cluster trend analysis of the population with no increase in anxiety scores (Anxiety.d. ≤ 0): H_anxiety_ = 0.8661411; (B) Cluster trend analysis of the population with no increase in depression scores (Depression.d.≤0): H_depression_ = 0.7695719; (C) Cluster effect analysis of the population with no increase in anxiety scores (Anxiety.d.≤0) average silhouette width = 0.33; (D) Cluster effect analysis of the population with no increase in depression scores (Depression.d.≤0) the average silhouette width = 0.26.

During the quarantine, we found that the number of people whose anxiety scores did not increase (Anxiety.d. ≤ 0) was 369, and the number of people whose depression scores did not increase (Depression.d. ≤ 0) was 338. The variables that affected Anxiety.d. and Depression.d. were included in the cluster analysis, respectively. When the H value was higher than 0.5, it meant that there was a trend of clustering in the data at a 90% confidence level (H_anxiety_ = 0.8661411; H_depression_ = 0.7695719) (Figures 2A,B). Using the k-means cluster analysis method, the two groups were clustered separately by selecting different k values, and the clinical significance of the clustering results was finally determined by dividing each group into 3 clusters, with each cluster in each group having its own characteristics. The average silhouette widths of both groups were > 0, which can be seen to have clustering accuracy (Figures 2C,D).

**Figure 3:**
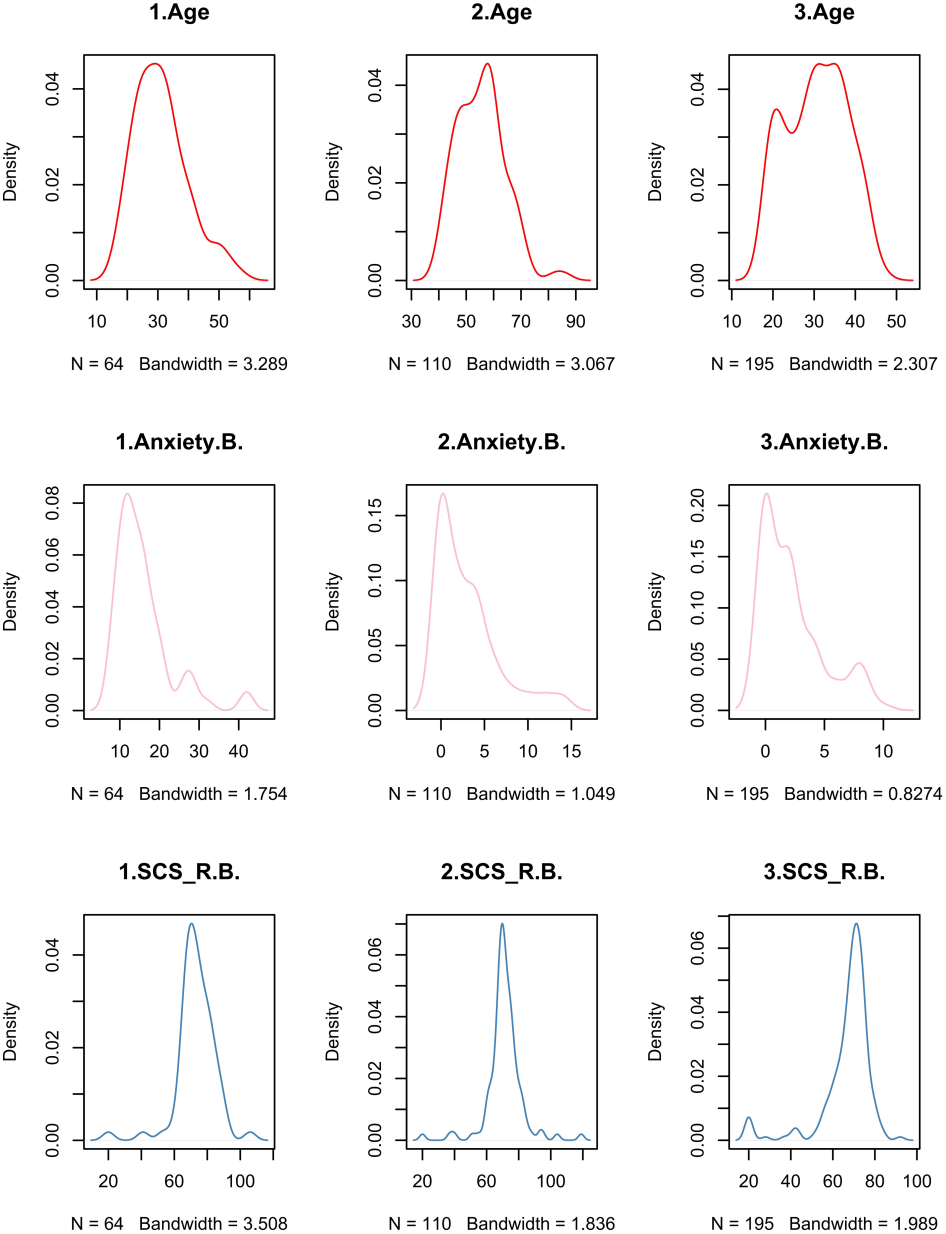
Characteristics of the 3 clusters of people whose anxiety scores did not increase during the quarantine. 1 = cluster1; 2 = cluster2; 3 = cluster3. Anxiety. B. = Pre-quarantine Anxiety Score; SCS_R.B. = Pre-quarantine Levels of Social Connectedness Scale-Revised.

**Figure 4:**
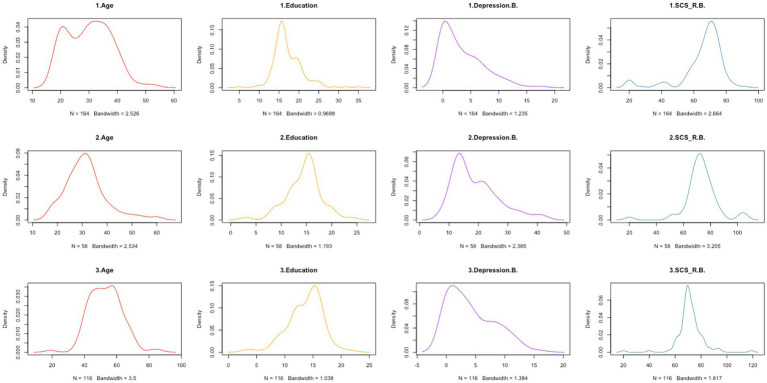
Characteristics of the 3 clusters of people whose depression scores did not increase during the quarantine. 1 = cluster1; 2 = cluster2; 3 = cluster3. Depression. B. = Pre-quarantine Depression Score; SCS_R.B. = Pre-quarantine Levels of Social Connectedness Scale-Revised.

The three clusters of the groups were characterized as follows (Figures 3, 4). After ANOVA and multiple comparison analysis, in the group (Anxiety.d. ≤ 0), in terms of age, cluster 2 was higher than cluster1 and cluster3, respectively; in terms of pre-quarantine anxiety scores, cluster1 was higher than cluster2 and cluster3, respectively; in terms of perceived social connectedness before quarantine, cluster3 was lower than cluster1 and cluster2, respectively (Supplementary Table S2). In the group (Depression.d. ≤ 0), in terms of age, cluster 3 was higher than cluster 1 and cluster 2, respectively; in terms of pre-isolation depression scores, cluster 2 was higher than cluster 1 and cluster 3, respectively; in terms of education, cluster1 was higher than cluster2 and cluster3, respectively; in terms of perceived social connectedness before quarantine, cluster1 was lower than cluster2 and cluster3, respectively (Supplementary Table S3).

### 3.5 Moderated mediation model

**Figure 5:**
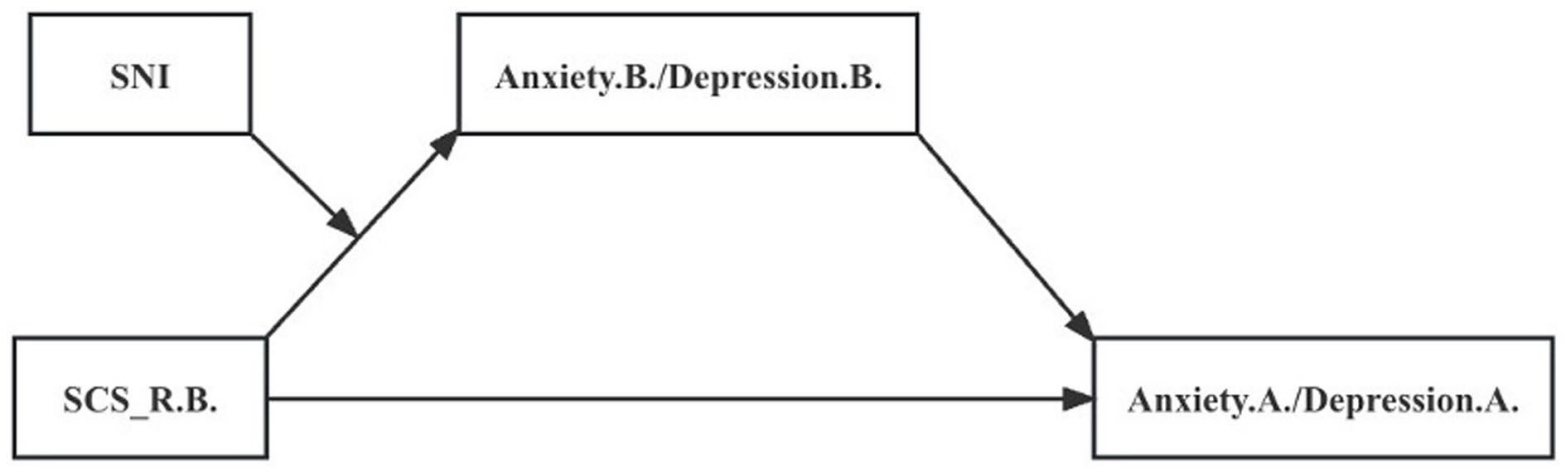
Moderated Mediation Model. The mediating role of mental states before quarantine and the moderating role of the score of SNI. SNI = Berkman-Syme social network index; SCS_R.B. = Pre-quarantine Levels of Social Connectedness Scale-Revised; Anxiety.B. = Pre-quarantine Anxiety Score; Depression. B. = Pre-quarantine Depression Score.

The degree of social connectedness affected not only pre-quarantine anxiety and depression status but also the status during the quarantine. We found that negative emotion before the quarantine was a potential candidate mediator, and research found that participants with higher baseline mental symptoms exhibited more severe symptoms after the blockade period (54). The size of social networks, another factor influencing mental health status, was associated with pre-quarantine anxiety and depression levels. Previous studies have also shown that social networks affect mental health, but we found no relationship between the size of social networks and changes in anxiety and depression resulting from quarantine. Besides, we found no significant differences in social connectedness scores under different sizes of social networks (Supplementary Table S4). We then hypothesized that anxiety and depression levels mediate between the degree of social connectedness before quarantine, and the anxiety and depression levels during the quarantine (Figure 5). The size of social networks acts as a moderator of the first half of the mediating pathway (the role of the degree of social connectedness on the pre-quarantine negative emotion). We speculated that the positive effect of the degree of subjective social connectedness on pre-quarantine anxiety and depression was stronger in individuals experiencing smaller social networks relative to those experiencing large social networks.

**Figure 6:**
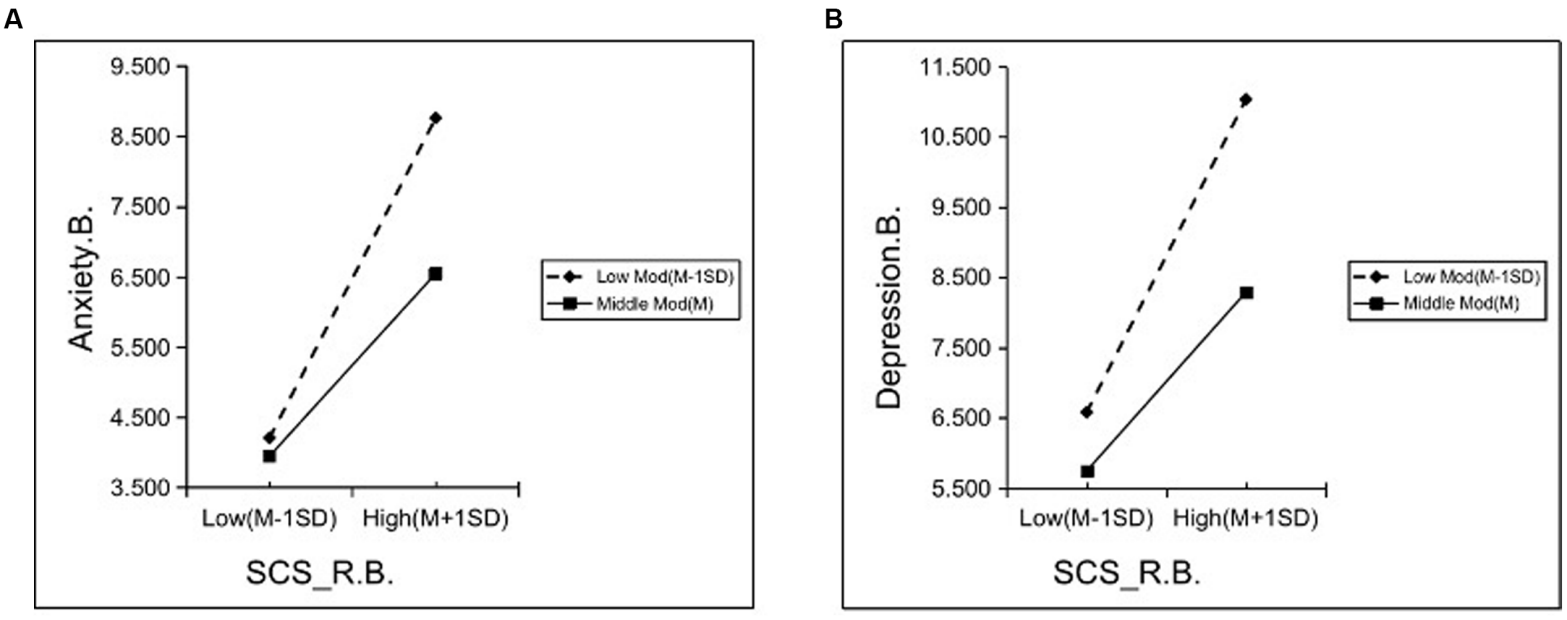
Simple slope analysis of the interaction between the degree of social connectedness and social isolation on pre-quarantine mental health status. (A) The interaction on pre-quarantine anxiety; (B) The interaction on pre-quarantine depression; SCS_R.B. = Pre-quarantine Levels of Social Connectedness Scale-Revised; Anxiety. B. = Pre-quarantine Anxiety Score; Depression. B. = Pre-quarantine Depression Score.

The mediating effect analysis with moderation (Table 3) showed a significant direct predictive effect of SCS_R.B. on Anxiety.A. and Depression.A. Further, Anxiety.B. and Depression.B. were included in the regression equation as mediating variables and SNI as a moderating variable, respectively. We found that SCS_R.B. had a significant positive predictive effect on Anxiety.B. and Depression. B. The upper and lower bounds of the boot 95% CI for the mediating effect of Anxiety.B. and Depression.B. did not include 0 when the SNI score was low and middle levels, indicating the presence of a mediating effect. Then, when SNI score was high level, the mediating effect did not exist. In addition, the interaction term of SCS_R.B. with SNI significantly and negatively predicted Anxiety.B. and Depression.B., indicating that the effect of SCS_R.B. on Anxiety.B. and Depression.B. was moderated by the degree of objective social isolation. In summary, the analysis showed that the mediating effect profile was inconsistent at different sizes of social networks, and there was a mediating effect with moderation (Table 4). To reveal how SNI moderates the effect of SCS_R.B. on Anxiety.B. and Depression.B., simple slope analysis plots were plotted for low and middle groupings based on the socres of SNI (Figure 6). The results showed that at low levels of SNI scores (the most socially isolated), SCS_R.B. significantly and positively predicted Anxiety.B. (b_simple_ = 0.390, *p* < 0.001), Depression.B. (b_simple_ = 0.381, *p* < 0.001), indicating that pre-quarantine social connectedness had a greater effect on anxiety and depression symptoms during quarantine, mediated by pre-quarantine anxiety and depressive symptoms when the mediating effects accounted for 66.0 and 58.3% of the respective total effects. The positive predictive effect on Anxiety.B. (b_simple_ = 0.223, *p* < 0.001) and Depression.B. (b_simple_ = 0.218, *p* < 0.001) remained significant but diminished when the SNI score was at middle levels, at which point the mediating effect accounted for 52.6 and 44.4% of the respective total effects. The result suggested that an increase in social networks would diminish the effect of the degree of social connectedness on pre-quarantine psychological status, which in turn would affect anxiety and depression levels during quarantine.

**Table 3:** Regression analysis of the mediating effect of SNI as moderator.

	Anxiety.B.		Anxiety.A.		Depression.B.		Depression.A.	
	*β*	*p*	*β*	*p*	*β*	*p*	*β*	*p*
SCS_R.B.	0.249	<0.001*	0.090	<0.001**	0.244	<0.001**	0.120	<0.001**
SNI	4.572	0.017**			3.904	0.085		
SCS_R.B.*SNI	−0.083	0.002*			−0.081	0.011*		
Age	−0.046	0.059	−0.047	0.013*	−0.046	0.118	−0.051	0.020*
Anxiety.B.			0.895	<0.001**				
Education					−0.142	0.114	0.162	0.024*
Depression.B.							0.880	<0.001**
*R* ^2^	0.105		0.590		0.108		0.606	
*F*	14.038		230.450		11.545		184.939	
*p*	<0.001**		<0.001**		<0.001**		<0.001**	

*p < 0.05 **p < 0.01. SNI=Berkman-Syme social network index; Anxiety.B. = pre-quarantine anxiety score; Depression.B. = pre-quarantine depression score; SCS_R.B. = pre-quarantine levels of social connectedness scale-revised; Boot95%CI = the lower and upper limits of the 95% interval for bootstrap sampling.

**Table 4:** Analysis of the moderating effect of SNI.

Mediator	Effect type	Level	SNI	Effect	SE/BootSE	95%CI /Boot95%CI	Explanatory power (%)
Anxiety.B.	Direct	–	–	0.090	0.021	0.048,0.132	–
	Indirect	Low (M-1SD)	0.659	0.175	0.077	0.017,0.319	66.0
		Middle (M)	1.672	0.100	0.042	0.015,0.178	52.6
		High (M + 1SD)	2.685	0.025	0.029	−0.030,0.084	0
Depression.B.	Direct	–	–	0.120	0.024	0.072,0.167	–
	Indirect	Low (M-1SD)	0.659	0.168	0.077	0.019,0.319	58.3
		Middle (M)	1.672	0.096	0.044	0.011,0.184	44.4
		High (M + 1SD)	2.685	0.024	0.035	−0.041,0.099	0

SNI=Berkman-Syme social network index; Anxiety.B. = pre-quarantine anxiety score; Depression.B. = pre-quarantine depression score. Boot95%CI = the lower and upper limits of the 95% interval for bootstrap sampling.

## 4 Discussion

The COVID-19 pandemic and the resulting quarantine measures posed additional psychological challenges to the population. Not only did the COVID-19 pandemic increase anxiety and depression symptoms in the population, but the isolation measures adopted by the mass pandemic also increased anxiety and depression symptoms, which is consistent with the findings of Verma et al. (55). Previous studies have demonstrated that enforced quarantine measures increase the prevalence rates of anxiety and depression (56, 57). Loneliness, as an unintended consequence of enforced quarantine, is associated with high rates of depression and anxiety (57). There were more females in our sample (69.28%), and females were more likely to experience anxiety and depressive symptoms than males (58). The small increase in depression and anxiety symptoms in our study may be because the study population was in the COVID-19 pandemic and inherently had higher levels of anxiety and depression than before the pandemic. In a study of 56,679 participants from 34 provinces in China, 27.9% had symptoms of depression, 31.6% had symptoms of anxiety, and 24.4% had symptoms of acute stress during the coronavirus disease 2019 (COVID-19) outbreak (59). Secondly, the population in this investigation was basically in the second week of the quarantine days, and previous studies have shown that the highest levels of anxiety and depression occurred during the first week of quarantine and symptoms began to decline in the second week (60). According to a survey, individuals’ mental health initially deteriorated during the COVID-19 pandemic, followed by stabilization as the pandemic persisted (61).

The study investigated the relationship between sociodemographic characteristics, degree of social connectedness, social networks, and changes in psychological status in people experiencing quarantine. Our sample was less older adult and predominantly middle-aged, which may explain why there was no significant change in subjective social connectedness in our study. Our findings are consistent with previous studies in that age and educational attainment are strongly correlated with depression symptoms (62, 63), and age is also associated with anxiety symptoms (63). We found that anxiety and depression scores before quarantine negatively predicted changes in anxiety and depression scores from quarantine measures, possibly because people with more anxiety and depression symptoms before isolation would show a decrease in anxiety and depression during quarantine (64). Another study also found that school closures during quarantine were beneficial in reducing depression and anxiety symptoms in adolescents (65).

Previous research has indicated that subjective social connectedness has an impact on the mental health of transgender youth during the pandemic, with social connectedness and social support being significant predictors of depression and anxiety severity (66). In a prospective study of adolescent mental health during COVID-19, social connectedness was found to be a protective factor for poor mental health during follow-up (67). In contrast to previous literature, we found that higher social connectedness was associated with greater anxiety and depression symptoms during the pandemic and with greater increases in anxiety and depression scores associated with isolation measures. There are several possible reasons why our findings differ from previous studies.

The prevalence of loneliness increased to 51% during the pandemic (68). The mean social connectedness scores of our population were lower than those previously reported in the literature, which may be related to higher levels of loneliness. Amrish et al. found that social connectedness can affect the quality of life and well-being through loneliness (69). This may be another pathway through which the level of social connectedness influences anxiety and depressive symptoms in our study. Different measurement instruments: the methods used to measure social connectedness, social networks, and mental health status can affect the results. The Multidimensional Scale of Perceived Social Support (MSPSS) is commonly used for scales of social relationship support in previous studies, and the GAD-7 and the PHQ-9 are commonly used as anxiety and depression measurement scales in previous studies. Sample differences: differences in the study population can affect the results. Our study was voluntary and web-based, and people with more severe symptoms and those who do not use the web may have been excluded. Timing of measurements: the timing of measurements can be critical, especially during a pandemic when environmental and stress factors can change rapidly. We observed no significant change in social connectedness scores during isolation, possibly because it was not the first-time participants had experienced a quarantine when we measured it and the population had been locked down for more than a week.

We did cluster analyses separately for those whose anxiety and depression scores did not increase, refining the population classification. Each group was classified into 3 clusters, further exposing the respective characteristics of the relevant variables in the different clusters. Based on this classification, more targeted measures can be taken to help improve the mental health of the population.

Our study combines subjective social connectedness and objective social networks to explore the role of changes in mental health brought about by quarantine. The results of the mediation analysis suggest that pre-quarantine negative emotions mediated the relationship between the degree of pre-quarantine social connectedness and negative emotions during the quarantine. The results of this study further confirm previous evidence that pre-quarantine mental state is a significant predictor of mental state during quarantine (54). The poorer mental health status of the population during the pandemic, despite being in a non-quarantine period, was a major psychiatric consequence of COVID-19 (32, 70). Social connectedness not only played a direct role in the changes in mental status brought about by quarantine but also influenced anxiety and depression symptoms during the quarantine by affecting emotional status before the pandemic. Results from moderated mediation analyses suggested that an increase in SNI scores (expansion of the size of social networks) would buffer the effect of the level of social connectedness on pre-quarantine anxiety and depression. Previous research has also shown that small size social networks are associated with more severe physical and psychological problems (71). Interventions, such as enhanced community support programs designed to improve digital literacy for social use, could help to reduce loneliness and increase social connectedness (69). Psychological interventions and community outreach, such as community college for the older adult, can be designed for this population with the aim of increasing their frequency of social contact and helping them reduce symptoms of anxiety and depression during the pandemic.

Our study excluded individuals with previous COVID-19 infection because COVID-19 infection may influence the development of psychiatric disorders, including depression (72) and the potential neurotrophic properties of the virus (31, 73–75). This facilitated the reduction of the effect of confounding factors in the study. However, this study has several limitations. First, our sample was biased: we studied people who voluntarily participated in an online survey, yet people with more severe symptoms and those who are not skilled in the use of the internet may tend not to participate in the study. Second, all information in this study was retrospective and self-reported, increasing the potential for reporting bias. Additionally, we did not collect vaccination information from our participants. Vaccination reduces the risk of severe COVID-19 illness, alleviates the fear of infection, and decreases the stressor, which is associated with a significantly lower risk of psychotic disorder (76). Finally, there is limited evidence available to replicate the results of this study, and further studies with larger sample sizes and more robust methods (e.g., prospective longitudinal studies) are needed to replicate the results of this study.

## 5 Conclusion

Despite its limitations, our study revealed that quarantine increases anxiety and depressive symptoms. Increasing age was a protective factor against declining anxiety and depression during the quarantine, but increasing education and social connectedness were risk factors for declining depressive symptoms. Cluster analysis was conducted for those whose anxiety and depressive symptoms did not aggravate during the quarantine, revealing three distinct clusters in both populations, each with its own characteristics. The results of the moderated mediation analysis suggested that people experiencing smaller social networks when perceiving the same level of social connectedness may tend to suffer more severe psychological distress during quarantine, prompting us to focus on the most socially isolated people (SNI = 0/1). Interventions, including psychological interventions and community outreach, can be designed for this population with the aim of increasing their frequency of social contact and helping them reduce symptoms of anxiety and depression during the pandemic.

## Data availability statement

The raw data supporting the conclusions of this article will be made available by the authors, without undue reservation.

## Ethics statement

The studies involving humans were approved by the West China Hospital of Sichuan University Biomedical Research Ethics Committee. The studies were conducted in accordance with the local legislation and institutional requirements. The participants provided their written informed consent to participate in this study.

## Author contributions

HL: Writing – original draft. DL: Data curation, Methodology, Writing – review & editing. QT: Methodology, Writing – review & editing. ZN: Methodology, Writing – review & editing. JX: Writing – review & editing. JL: Writing – review & editing.
