# Supplementary material for: The combined impact of social networks and connectedness on anxiety, stress, and depression during COVID-19 quarantine: a retrospective observational study
Source: Front Public Health. 2023 Dec 19;11:1298693. doi: 10.3389/fpubh.2023.1298693 (PMC10758457; doi:10.3389/fpubh.2023.1298693)
Supplement: Supplementary file 1 [file Table_1.docx]

Table S1. Changes in mental state and social connectedness before and during the quarantine.

| **Variables** | **paired (M ± SD)** | | **d(During-Before)** | ***t*** | ***p*** |
| --- | --- | --- | --- | --- | --- |
|  | **Before** | **During** |  |  |  |
| Stress | 8.73±8.81 | 9.08±10.29 | 0.36 | 1.161 | 0.246 |
| Anxiety | 5.28±6.79 | 5.94±8.43 | 0.66 | 2.605 | 0.009** |
| Depression | 7.05±8.07 | 7.71±9.70 | 0.66 | 2.302 | 0.022* |
| SCS_R | 70.20±11.68 | 70.95±12.23 | 0.74 | 1.875 | 0.061 |

Note. **p*<0.05, ***p*<0.01; SCS_R: Social Connectedness Scale-Revised.
