# Supplementary material for: The combined impact of social networks and connectedness on anxiety, stress, and depression during COVID-19 quarantine: a retrospective observational study
Source: Front Public Health. 2023 Dec 19;11:1298693. doi: 10.3389/fpubh.2023.1298693 (PMC10758457; doi:10.3389/fpubh.2023.1298693)
Supplement: Supplementary file 2 [file Table_2.docx]

|  | **Clustering categories** | | | ***F*** | ***p*** | **Scheffe** |
| --- | --- | --- | --- | --- | --- | --- |
|  | **Cluster 1(*n*=64)** | **Cluster 2(*n*=110)** | **Cluster 3(*n*=195)** |  |  |  |
| Age | 31.09±8.655 | 55.42±8.723 | 30.59±7.360 | 366.658 | <0.001** | cluster 2>cluster 1; cluster 2>cluster 3 |
| Anxiety.B. | 15.84±7.227 | 2.96±3.550 | 2.26±2.639 | 287.121 | <0.001** | cluster 1>cluster 2; cluster 1>cluster 3 |
| SCS_R.B. | 73.14±11.658 | 71.16±11.027 | 66.76±12.184 | 9.310 | <0.001** | cluster 1>cluster 3; cluster 2>cluster 3 |

Table S2. Results of the cluster category ANOVA difference comparison and multiple comparison analysis for the person without increasing anxiety scores.

Note. Anxiety.B.=Pre-quarantine Anxiety Score; SCS_R.B.=Pre-quarantiganzhi ne Levels of Social Connectedness Scale-Revised; **p*<0.05, ***p*<0.01.
