# Supplementary material for: The combined impact of social networks and connectedness on anxiety, stress, and depression during COVID-19 quarantine: a retrospective observational study
Source: Front Public Health. 2023 Dec 19;11:1298693. doi: 10.3389/fpubh.2023.1298693 (PMC10758457; doi:10.3389/fpubh.2023.1298693)
Supplement: Supplementary file 3 [file Table_3.docx]

**Table S3. Results of the cluster category ANOVA difference comparison and multiple comparison analysis for the person without increasing depression scores.**

|  | **Clustering categories** | | | ***F*** | ***p*** | **Scheffe** |
| --- | --- | --- | --- | --- | --- | --- |
|  | **Cluster 1(*n*=164)** | **Cluster 2(*n*=58)** | **Cluster 3(*n*=116)** |  |  |  |
| Age | 30.02±7.78 | 31.59±8.39 | 53.11±10.06 | 257.727 | <0.001** | cluster 3>cluster 1; cluster 3>cluster 2 |
| Education | 17.23±3.76 | 14.52±3.48 | 13.66±3.10 | 38.262 | <0.001** | cluster 1>cluster 2; cluster 1>cluster 3 |
| Depression.B. | 3.48±3.80 | 19.07±7.88 | 4.26±3.98 | 244.047 | <0.001** | cluster 2>cluster 1; cluster 2>cluster 3 |
| SCS_R.B. | 65.68±13.36 | 73.21±12.37 | 71.53±9.94 | 12.185 | <0.001** | cluster 2>cluster 1; cluster 3>cluster 1 |

Note. Depression.B.=Pre-quarantine Depression Score; SCS_R.B.=Pre-quarantine Levels of Social Connectedness Scale-Revised; * *p*<0.05; ** *p*<0.01.
