# Supplementary material for: The combined impact of social networks and connectedness on anxiety, stress, and depression during COVID-19 quarantine: a retrospective observational study
Source: Front Public Health. 2023 Dec 19;11:1298693. doi: 10.3389/fpubh.2023.1298693 (PMC10758457; doi:10.3389/fpubh.2023.1298693)
Supplement: Supplementary file 4 [file Table_4.docx]

Table S4. ANOVA for social connectedness at different sizes of social networks.

|  | **SNI (M ± SD)** | | | | | ***F*** | ***p*** |
| --- | --- | --- | --- | --- | --- | --- | --- |
|  | **0.0(*n*=54)** | **1.0(*n*=173)** | **2.0(*n*=155)** | **3.0(*n*=84)** | **4.0(*n*=19)** |  |  |
| SCS_R.B | 71.74±7.08 | 70.03±12.44 | 70.16±10.56 | 69.69±14.77 | 70.00±8.57 | 0.284 | 0.889 |
| SCS_R.A | 72.11±10.70 | 71.10±12.90 | 70.80±10.37 | 70.06±15.54 | 71.37±7.06 | 0.249 | 0.910 |
|  | | | | | | | |

Note. SCS_R.B.=Pre-quarantine Levels of Social Connectedness Scale-Revised; SCS_R.A.=Levels of Social Connectedness Scale-Revised During the Quarantine ;
